# Community Mobilisation for Human Sample Collection in Sensitive Communities: Experiences from Granular Mapping of Schistosomiasis and Soil-Transmitted Helminths in Ekiti State, South West, Nigeria

**DOI:** 10.3390/tropicalmed9110255

**Published:** 2024-10-25

**Authors:** Temitope Agbana, Omolade Omotade, Moses Aderogba, David Bell, Jacob Solomon, Saheed Animashaun, Peace Alabi, Oladimeji Ajayi, Adebowale Akinwumi, Samuel Popoola, Alex Bunda, Jan-Carel Diehl, Gleb Vdovine, Louise Makau-Barasa

**Affiliations:** 1 AiDx Medical Bv, 2641 KM Pijnacker, The Netherlands; 2 The Ending Neglected Diseases (END) Fund, New York, NY 10016, USA; 3 Independent Consultant, Lake Jackson, TX 77566, USA; 4 The Neglected Tropical Diseases (NTD) Division, Federal Ministry of Health, Abuja 900242, Nigeria; 5 The Neglected Tropical Diseases (NTD) Division, Ekiti State Ministry of Health, Ado Ekiti 360101, Nigeria; 6 Department of Community Medicine, Faculty of Clinical Science, College of Medicine, Ekiti State University, Ado Ekiti 362103, Nigeria; 7 Sustainable Design Engineering, Delft University of Technology, 2628 CE Delft, The Netherlands

**Keywords:** community mobilisation, sample collection, advocacy, schistosomiasis mapping, STH, Ekiti, South West

## Abstract

Community mobilisation is a vital process for raising awareness and increasing participation in healthcare interventions, research, and programmes that require human sample collection and mass management. In this report, we present the community mobilisation approach undertaken for the implementation of the operational mapping and assessment of granular schistosomiasis and soil-transmitted helminths in Ekiti State, Nigeria. The mobilisation was conducted in 177 communities/wards of the 16 local government areas. A total of 15,340 urine and stool samples were collected in 34 days. The efficacy and success of the strategy were evaluated through the following three performance metrics: community compliance rate, the participant response rate at the community level, and the overall compliance response rate of the four most sensitive LGAs. Community compliance was 93.7% as sample collection was denied in nine communities and two other communities demanded the return of the collected samples despite our mobilisation effort because of cultural bias and myths that connect the collection of stool and urine samples to ritual activities in the local context. The participant response rate at the community level was 86.7%. Three of the four sensitive LGAs (based on previous assessment programmes) demonstrated satisfactory compliance rates of 100%, while a response rate of 64.0% was computed for one of the LGAs. We believe our approach contributed to effective community mobilisation and awareness and that the developed model has the potential to improve participation rates in large healthcare assessments and intervention programmes.

## 1. Introduction

Schistosomiasis, a neglected tropical disease (NTD) caused by parasitic flatworms of the genus Schistosoma, poses a persistent threat to public health in various regions. This helminth infection infects approximately 250 million people, particularly those living in low- and middle-income African countries [1,2,3]; it is a prevalent NTD in sub-Saharan Africa. Nigeria has the highest number of infected individuals, with approximately 29 million reported cases and an additional 101 million individuals at risk of infection [4,5,6,7,8]. The transmission of this disease occurs through exposure to freshwater containing cercariae, which is the infective larval stage that is released from intermediate host snails [9].

There are two major forms of schistosomiasis—intestinal (due to *Schistosoma mansoni* and *Schistosoma japonicum*) and urogenital (predominantly due to *Schistosoma haematobium*). Common signs and symptoms of urogenital *S. haematobium* include a swollen belly, blood in the urine, stunted growth, cognitive impairment in children, and infertility among adults of childbearing age. Notably, *S. haematobium* is also associated with squamous cell carcinoma of the bladder, adding a critical dimension to its pathogenicity [6,7,10].

Soil-transmitted helminths (STHs) constitute a group of parasitic nematodes affecting humans; they are primarily prevalent in tropical and subtropical regions, particularly in low- and middle-income countries [11]. In terms of morbidity, infections caused by soil-transmitted helminths are considered among the most significant neglected tropical diseases, with nearly one billion individuals still harbouring at least one species [12]. The primary soil-transmitted helminths affecting humans include roundworms (*Ascaris lumbricoides*), whipworms (*Trichuris trichiura*), and hookworms (*Necator americanus* and *Ancylostoma duodenale*) [13].

In 2020, the World Health Organization (WHO) launched a strategic initiative aimed at eradicating schistosomiasis as a public health concern by 2030. This comprehensive plan involves a multi-faceted approach, including ensuring access to improved drinking water, enhancing sanitation, providing hygiene education, managing the environment, controlling snail populations, administering preventive chemotherapy to vulnerable groups, and the use of diagnostic tests for the assessment of schistosomiasis [14,15].

Within the specific context of Ekiti state, Nigeria, initiatives to combat schistosomiasis employ diverse strategies, such as the promotion of improved sanitation, the dissemination of health education, and the implementation of annual rounds of mass drug administration (MDA) based on the prevalence of the disease in each implementing units using praziquantel [16,17,18,19]. In Ekiti state, MDA has been ongoing since 2015 with annual treatments being delivered to populations based on endemicity levels and the WHO guideline on the control and elimination of human schistosomiasis treatment guideline. Currently, the operational evaluation and impact assessment of the MDA programmes largely depends on the collection and microscopy analysis of urine samples and faecal matters. This approach complies with WHO guidelines on the assessment of schistosomiasis that are included in the aforementioned WHO guideline. The samples are examined for the presence of parasite eggs, while the infection load estimates provide insight into the efficacy of the treatment.

In this study, we report the awareness campaign, community mobilisation strategies, and approaches used amidst misinformation about sample collection during the granular operational assessment of schistosomiasis and STH in Ekiti state in Southwest Nigeria in the year 2023. The primary objective of the granular operational assessment was to determine the prevalence and intensity of *S. haematobium*, *S. mansoni*, and STH at the ward administrative level.

## 2. Materials and Methods

### 2.1. Collaborative Strategy Development for Community Mobilisation

A collaborative strategy meeting to develop a strategic approach to community mobilisation was convened by the Ekiti State Ministry of Health and Human Services—Neglected Tropical Disease Department. The mobilisation approach identified stakeholders at different arms and levels of government with the potential to engage the community and address potential fears and concerns due to the sample collection and overall objective of the study. Community stakeholders were identified as trusted local community members that could potentially facilitate community acceptance of the project and raise community ownership and participation. This proposed involvement intended to ensure community members were provided with the correct information about the operational mapping exercise and its communal benefits. The use of image-based awareness campaign techniques as proposed by Makau-Barasa et al. [18] was also adopted. During the strategy meeting, critical hotspots of potential resistance to the project based on the previous mapping were identified. Four challenging local government areas (LGAs) were identified—Ikere, Ise Orun, Ekiti Southwest, and Emure. These areas were earmarked for intense awareness campaigns and community mobilisation. These LGAs were also strategically reserved as the last regions for sample collection as success in other communities could be used as part of the mobilisation strategies in these LGAs. Training plans, community media outreach strategies, and advocacy visits to religious, community, and traditional leaders and their institutions were incorporated into the strategy. Another important stakeholder of interest was the Ekiti State University Teaching Hospital. The involvement of the department of Community Medicine also enhanced trust and increased community participation.

### 2.2. Community Mobilisation

A quick sampling technique was used to categorise the communities based on their envisaged preference. A priori knowledge of community preferences, beliefs, culture, and sensitivity enhanced data collection and entry during the study. The mobilisation protocol for local community leaders was adapted for the following categories:

#### 2.2.1. Mobilisation of Community Leaders

Community leaders were subdivided into four main categories, as follows: (1) political actors; (2) traditional leaders; (3) religious leaders; and (4) market and community youth leaders. Traditional palaces, mosques, churches, and town halls were strategically visited, and leaders informed utilising an image-based information approach showing the target parasites, as reported by Makau-Barasa et al. [18].

#### 2.2.2. Mobilisation of School Heads and Parents Teachers Associations

A school mobilisation protocol included advocacy to the school heads and the chairpersons of the Parents Teachers Associations (PTAs). To gain the buy-in of the target leaders, Local Government Education Secretaries were included in the mobilisation team. School mobilisation was carried out one week after the intensive community mobilisation. Training was then organised for all school heads within a community to ensure that the entire community was well informed. Parental consent forms were then issued to the head teachers for distribution to their students.

#### 2.2.3. Community Publicity and Awareness Campaign

**Figure 1:**
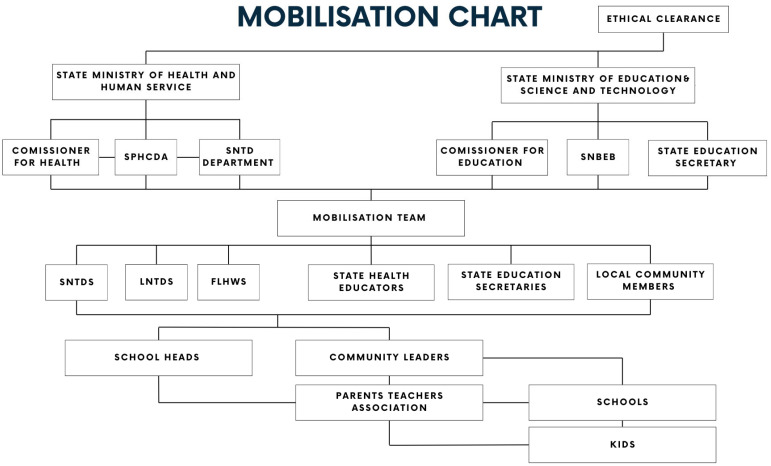
Mobilisation chart.

An extensive awareness campaign was conducted in town squares and market places. The mobilisation team planned the advocacy outreach to secure the collaboration of community market and youth leaders. Women market leaders were identified, and they were trained to reach out to parents of the target school age children. Figure 1 below shows the chart of mobilisation for advocacy and sample collection.

## 3. Results

Based on the reference standard sample collection of 50 samples per community, 17,700 urine and stool samples were expected [20]. A total of 15,340 urine and stool samples were collected, and the sample collection process occurred over 34 days. 

The participant response rate at the community level was 86.7%, as shown in Table 1. The participant response rate is computed based on the ratio of the total number of actual participants (7670) to the total number of expected participants (8850).

More than an 80% compliance rate was achieved in 11 out of the 16 LGAs. The remaining five LGAs demonstrated a >50% compliance rate.

The four sensitive LGAs—Ikere, Ise Orun, Ekiti Southwest, and Emure—interestingly demonstrated satisfactory compliance rates of 100%, 100%, 100%, and 64%, respectively, as shown in Table 2.

The overall community compliance rate was 93%. An exceptional 100% compliance was observed in the following LGAs: Ekiti East, Ilejemeje, Ikere, Ekiti Southwest, Ise/Orun, and Ado Ekiti. The 100% compliance in the highlighted communities emphasises the need for clearly planned and effective mobilisation strategies that should be designed to include both high- and low-level stakeholders in the community of interest. Sample collection was successful in 166 out of the 177 communities in the state. Nine communities declined participation in the project and two other communities demanded the return of the collected samples despite our mobilisation effort because of cultural bias and myths that connect collection of stool and urine samples to ritual activities in the local context.

## 4. Discussion

A deep-seated mistrust of health research and a reluctance to participate in surveys involving human samples significantly impact operational assessments that could guide policies and decisions in control and elimination programmes. Consequently, the acquisition of human samples (urine and stool samples) for large-scale operational assessments of schistosomiasis in Ekiti state was a challenge.

One significant incident highlighting this challenge occurred in the second quarter of 2013 in Ekiti State, Nigeria, as is understood from a series of interviews carried out with staff at the Ekiti State Primary Health Care Development Agency. The incident is reported as follows:

Representatives from the Federal Ministry of Health with the research objective of assessing the prevalence of schistosomiasis selected five schools from each of the 16 LGAs for sample collection and analysis. The head teachers at the selected schools were adequately informed of the assessment programme. Ethical protocols and procedures were duly followed before sample collection commenced in the selected LGAs.

The project was discontinued when the rumoured circulation of the myth or belief that collected urine and stool samples would be used for ritual purposes provoked vehement opposition to the project. In some communities, parents demanded the return of all collected samples. The delayed return of the samples triggered a massive state-wide protest. Some other communities agreed to participate only if the collected samples were processed on site. Based on this agreement, the assessment was re-conducted and concluded in 2014. The on-site sample collection created a serious logistic problem. According to the information obtained from Ekiti state NTD department of the Ministry of Health, no extensive impact assessment of schistosomiasis-related research has been implemented in Ekiti state since 2014.

Community fears, misconceptions, myths, concerns, and suspicions about human sample collection and use are not peculiar to Ekiti state alone. Other similar community misconceptions have been reported in the literature [10,21].

Therefore, community mobilisation remains an essential aspect of community health programmes. The importance of clear communication and partnerships with all stakeholders at all levels, as demonstrated in this study, cannot be overemphasised. For this assessment, a top–bottom and bottom-up approach was used. The top–bottom approach includes the involvement of high-level stakeholders in the state ministries of health and education to disseminate information that would support and enhance community acceptance. This approach established the necessary trust needed to validate the integrity, safety, and benefit of the project to the community members. The bottom-up method involves the use of front-line healthcare workers to enhance community participation.

The familiarity between the frontline workers and the community likely increased the success of sample collection. Mass awareness and mobilisation may have diminished fears, mistrust, prevailing rumours, myths, and misconceptions associated with the collections of intimate samples such as stool, urine, and blood.

Our proposed strategy facilitated the successful collection of 15,340 urine and stool samples from school age children (5–14 years) with parental consent. The achieved participation and compliance rate reported in this report demonstrates the promising potential of our proposed mobilisation strategy for health-related research and campaigns in sensitive communities.

Despite various mobilisation strategies, certain parents openly expressed their refusal for their children to participate in the assessment project. Some children returned unsigned consent forms; some parents withdrew their children from school during the sample collection exercise, while others prevented the sample collection process. All these practical issues account for the 86.7% response rate of participants estimated at the community level.

While a 100% response rate was recorded in some urban communities, community mobilisation and sample collection were most challenging in rural low-density areas. Potential impediments include the lack of access to state radio and television media where awareness messages about the exercise were disseminated, as well as a low level of outreach to community members who were away from their homes during the day.

Overall, a total of 11 communities were unwilling to participate in the exercise. Community members in 9 of these 11 communities made their intentions clear during community mobilisation, asserting their refusal to allow the collection of samples from their children.

The demand for the return of the samples that were collected and transported to the laboratory by parents in two communities was a serious challenge. Parents in those communities suddenly became agitated and staged a protest to demand the return of the collected samples despite their previous approval. The frontline healthcare workers prevented the further escalation of the situation by appealing for the intervention of the community leaders. The situation was resolved, and samples collected from the community were returned accordingly. These multifaceted challenges underscore the intricate nature of conducting health assessments within communities marked by deep-seated mistrust and resistance.

The 2013/2014 mobilisation strategy mobilised the involvement of the school authority. However, high-level stakeholder engagement, community mobilisation, Parent Teachers Association awareness, and mobilisation were excluded. Aside from complementing the identified gaps in the previous survey, our proposed mobilisation strategy sought to strengthen the Ekiti State Ministry of Health NTD Department to take ownership of the project and design a top–bottom, bottom–top approach for effective awareness campaigns and mobilisation. The local knowledge of practical challenges that are peculiar to each community enabled the design of a well-thought-through strategy that identified and engaged the right stakeholders for effective project dissemination at all levels. Allowing local context ownership of the project was critical to the success of the project.

To further improve the participation of other communities, we suggest a multiple iteration awareness campaign in the local community that will involve local political actors, government officials, and the State Ministry of Health. Discussion must be evidence-based. The results, community impact, and benefits derived from previous projects should be clearly presented to sensitise and stimulate participation.

To address the challenges identified in this schistosomiasis assessment and to establish a foundation for sustainable and community-driven health initiatives, we recommend the following: A clear community feedback mechanism, in which community members who gave their samples should receive timely and comprehensible feedback on the outcomes of the assessment. This could ensure a two-way communication channel, foster trust, and reinforce the notion that community participation yields tangible and meaningful results. Feedback sessions can be organised at community centres, involving local leaders to enhance the credibility of the information shared. We strongly recommend the use of the image-based awareness campaign method, as reported by Makau-Barasa et al. [18]. The use of images as part of the IEC material would enable the community members to appreciate the need for the assessment and the potential health benefit for their children and the selected community.

### Limitations of This Study

The limitation of this study is that the contribution of each specific activity (media campaign, engagement of community and religious leaders, involvement of parent-teacher association, etc.) to the whole strategy could not be estimated. Hence, the effectiveness of each approach in our developed model could not be quantified.

## 5. Conclusions

After the comprehensive design and implementation of community mobilisation for schistosomiasis and STH sampling, an 86.67% response rate was achieved in a set of communities that were previously refractory to these programmes

Successful mobilisation was achieved after the relevant stakeholders were involved and continuously engaged at the state and community levels. Regular consultation and several feedback iterations during the awareness campaign, community mobilisation, and sample collection process appear to have been successful.

From the lessons and practises learned from the granular schistosomiasis assessment project’s community mobilisation approach, other healthcare assessment programmes and large community-based outreach events can significantly benefit by improving and applying the outlined techniques.

## Figures and Tables

**Table 1:** Percentage compliance for all the 16 LGAs in Ekiti state.

LGA	Total Number of Wards	Total Number of Expected Participants	Total Number of Actual Participants	Average Number of Participants	Percentage Compliance (%)
Ikole	12	600	527	43.9	87.8
Oye	12	600	418	34.8	70.7
Emure	10	500	320	32.0	64.0
Ado	13	650	658	50.6	100.0
Ikere	11	550	550	50.0	100.0
Ekiti East	12	600	618	51.5	100.0
Moba	11	550	420	38.2	76.4
Ilejemeje	10	500	546	54.6	100.0
Ekiti Southwest	11	550	556	50.5	100.0
Ise/Orun	10	500	517	51.7	100.0
Ekiti West	11	550	469	42.6	85.3
Irepodun/Ifelodun	11	550	396	36.0	72.0
Efon	10	500	416	41.6	83.2
Ijero	12	600	303	25.3	50.5
Gbonyin	10	500	447	44.7	89.4
Ido/Osi	11	550	509	46.2	92.5
**Total Percentage Compliance Across the 16 LGAs**					**86.7**

**Table 2:** Percentage compliance of the four sensitive LGAs.

LGA	Total Number of Wards	Total Number of Expected Participants	Total Number of Actual Participants	Average Number of Participants	Percentage Compliance (%)
Emure	10	500	320	32.0	64.0
Ikere	11	550	550	50.0	100.0
Ekiti Southwest	11	550	556	50.5	100.0
Ise/Orun	10	500	517	51.7	100.0

## Data Availability

The datasets generated and analysed during the current study are not publicly available, but are available from the corresponding author upon reasonable request.
